# Molecular Mechanisms of Skatole-Induced Inflammatory Responses in Intestinal Epithelial Caco-2 Cells: Implications for Colorectal Cancer and Inflammatory Bowel Disease

**DOI:** 10.3390/cells13201730

**Published:** 2024-10-18

**Authors:** Katsunori Ishii, Kazuma Naito, Dai Tanaka, Yoshihito Koto, Koichi Kurata, Hidehisa Shimizu

**Affiliations:** 1 Graduate School of Natural Science and Technology, Shimane University, 1060 Nishikawatsu-Cho, Matsue 690-8504, Shimane, Japan; 2 Graduate School of Life and Environmental Science, Shimane University, 1060 Nishikawatsu-Cho, Matsue 690-8504, Shimane, Japan; 3 Department of Life Science and Biotechnology, Shimane University, 1060 Nishikawatsu-Cho, Matsue 690-8504, Shimane, Japan; 4 The United Graduate School of Agricultural Sciences, Tottori University, 4-101 Koyama-Minami, Tottori 680-8553, Tottori, Japan; 5 Estuary Research Center, Shimane University, 1060 Nishikawatsu-Cho, Matsue 690-8504, Shimane, Japan; 6 Interdisciplinary Center for Science Research, Shimane University, 1060 Nishikawatsu-Cho, Matsue 690-8504, Shimane, Japan; 7 Institute of Agricultural and Life Sciences, Academic Assembly, Shimane University, 1060 Nishikawatsu-Cho, Matsue 690-8504, Shimane, Japan

**Keywords:** inflammation, tryptophan metabolites, indole derivative, gut microbiota, intestinal epithelial cells

## Abstract

Inflammatory cytokines, such as tumor necrosis factor-α (TNF-α) and interleukin-6 (IL-6), in intestinal epithelial cells significantly contribute to inflammatory bowel disease (IBD) and colorectal cancer (CRC). Given our previous findings that TNF-α is upregulated in intestinal epithelial Caco-2 cells induced by skatole, a tryptophan-derived gut microbiota metabolite, the present study aimed to explore the relationship between skatole and IL-6, alongside TNF-α. Skatole elevated the promoter activity of IL-6 as well as TNF-α, and increased IL-6 mRNA expression and protein secretion. In addition to activating NF-κB, the NF-κB inhibitor BAY 11-7082 reduced skatole-induced cell survival and the mRNA expression of IL-6 and TNF-α. NF-κB activation was attenuated by the extracellular signal-regulated kinase (ERK) pathway inhibitor U0126 and the p38 inhibitor SB203580, but not by the c-Jun N-terminal kinase (JNK) inhibitor SP600125. U126 and SB203580 also decreased the skatole-induced increase in IL-6 expression. When skatole-induced AhR activation was inhibited by CH223191, in addition to promoting NF-κB activation, IL-6 expression was enhanced in a manner similar to that previously reported for TNF-α. Taken together, these results suggest that skatole-elicited NF-κB activation induces IL-6 and TNF-α expression, although AhR activation partially suppresses this process. The ability of skatole to increase the expression of IL-6 and TNF-α may significantly affect the development and progression of these diseases. Moreover, the balance between NF-κB and AhR activation appears to govern the skatole-induced increases in IL-6 and TNF-α expression. Therefore, the present findings provide new insights into the mechanisms linking tryptophan-derived gut microbiota metabolites with colorectal disease.

## 1. Introduction

Colorectal cancer (CRC) represents a significant global public health concern, as evidenced by the diagnosis of approximately 1.8 million individuals with the disease in 2018, resulting in 881,000 deaths and accounting for nearly 10% of all cancer diagnoses and fatalities [1]. CRC represents the third most prevalent cancer in terms of incidence and ranks second in terms of cancer-related mortality [1,2]. Projections indicate that, by 2030, the incidence of CRC cases and related deaths will exceed 2.2 million and 1.1 million, respectively [2,3]. The incidence of CRC is often correlated with socioeconomic development, increasing in parallel with the Human Development Index in rapidly developing countries [4,5]. One of the primary factors contributing to the development and progression of CRC is chronic inflammation of the colorectal tissue. For instance, obesity has been identified as a major risk factor for CRC and is associated with its development [6,7,8], a mechanism that has been implicated in diet-induced colitis due to the consumption of high-fat diets [8,9,10,11]. Furthermore, individuals diagnosed with inflammatory bowel disease (IBD), including ulcerative colitis (UC) and Crohn’s disease (CD), who experience chronic inflammation of the colon are recognized to have a significantly elevated risk of developing CRC [2,12,13,14,15,16].

Numerous signaling pathways are known to be disrupted during intestinal inflammation [17,18,19]. Intestinal epithelial cells are considered a major source of the pro-inflammatory molecule interleukin-6 (IL-6) in IBD [20]. The increase in IL-6 is more pronounced in patients with CD than in patients with UC [21,22,23]; the physiological plasma concentration of IL-6 is approximately 1.6 pg/mL but may elevate to 32.7 ng/mL in patients with CD [21,22,24]. Elevated IL-6 levels during intestinal inflammation lead to epithelial cell injury and chronic inflammation [25]. This process compromises the permeability of the intestinal epithelial barrier, resulting in cation leakage, which is a hallmark of exudative diarrhea in patients with IBD [26,27,28]. The serum concentration of IL-6 increases during the exacerbation phase of IBD and decreases during the remission phase of IBD [20,29]. Additionally, the severity of inflammatory alterations in the intestinal tract correlates with IL-6 levels [29]. A study in mice showed that IL-6 contributes to the development of colitis-associated cancer [30] and plays a crucial role in both the onset and progression of CRC in individuals with colitis-associated cancer [20,31]. Furthermore, overexpression of IL-6 has been observed in CRC tissues [19,32,33,34,35], and increased Il-6 expression has also been detected in epithelial cells of CRC [36,37]. Consistent with these findings, patients with CRC exhibit significantly elevated serum IL-6 levels compared to healthy individuals, and these levels demonstrate a correlation with tumor size, stage, metastasis, and survival [19,35,38,39,40]. Moreover, presurgical serum IL-6 levels greater than 10 pg/mL are predictive of diminished survival in patients with CRC, independent of the tumor site, grade, and stage [35,41,42]. Patients with CRC exhibiting lower IL-6 expression in their initial stages show extended periods without disease recurrence [42,43]. Conversely, higher IL-6 expression correlates with more advanced CRC stages and shorter patient survival times [42,44].

Tumor necrosis factor-α (TNF-α), a member of the extensive superfamily of type II transmembrane proteins, is a ligand that contributes to the persistent inflammation associated with both IBD and CRC. Elevated TNF-α levels have been recognized in both the serum and feces of patients with IBD as well as in experimental models of intestinal inflammation [45,46,47]. Moreover, pharmacological agents that inhibit TNF-α activity have demonstrated efficacy in treating IBD [48,49,50,51]. Increased TNF-α concentration is associated with CRC progression [52]. Furthermore, this cytokine plays a significant role in promoting the invasion and metastasis of CRC by triggering epithelial–mesenchymal transition [53]. Increased TNF-α expression is linked to more progressive stages of CRC [54] and the recurrence of tumors in patients with CRC exhibiting metastases [55].

The regulation of IL-6 and TNF-α expression, including that in intestinal epithelial Caco-2 cells, predominantly involves the activation of NF-κB, which comprises a dimer of p50 and p65 (RelA) [35,42,56,57,58,59]. NF-κB p65 is a transcriptional activation subunit containing several phosphorylated serine residues. Phosphorylation of serine 276 of NF-κB p65 is required for enhanced NF-κB transcriptional activity, although it does not affect nuclear translocation or DNA binding activity [60]. This is attributed to the fact that binding to CREB-binding protein (CBP)/p300 is essential for the augmentation of the transcriptional activity of NF-κB [61], and the interaction between CBP/p300 and NF-κB p65 necessitates phosphorylation of serine 276 of NF-κB p65 [62,63]. Consequently, phosphorylation of serine 276 of NF-κB p65 serves as an indicator of elevated transcriptional activity of NF-κB.

Metabolites derived from tryptophan in food proteins, particularly indole derivatives synthesized by the gut microbiota, are strongly linked to the onset and progression of noninfectious disorders [64,65,66,67]. Our previous research indicated that indoxyl sulfate, an indole derivative that accumulates in the blood during the progression of chronic kidney disease, contributes to CRC exacerbation via the activation of AhR, which is recognized as its receptor [68]. In contrast, indole-3-acetic acid (IAA), an indole derivative structurally similar to indoxyl sulfate, acts as an AhR ligand in intestinal epithelial Caco-2 cells [69]. However, it suppresses the growth of these cells and downregulates TNF-α through a mechanism that is independent of AhR [70,71].

The present study primarily focused on skatole, a compound produced by the gut microbiota from IAA. Typically, healthy individuals exhibit fecal skatole concentrations ranging from 0 to 5 μg/g, with some reports indicating levels of approximately 35 μg/g [72,73,74]. However, skatole levels rise in the large intestine of individuals who consume high amounts of animal protein [73,74]. This compound increases to approximately 100 μg/g in patients with compromised digestive function in the intestinal tract and in those with CRC [72,73,74]. Consuming high amounts of protein from animal sources has been linked to heightened risks of developing and worsening IBD, including UC and CD [75,76], as well as CRC [77]. These findings suggest that skatole may be a potential regulator of chronic inflammation associated with IBD and CRC. Indeed, our previous research indicated that, in addition to leading to increased CYP1A1 expression through the activation of AhR [78], skatole upregulates TNF-α expression, and this response is mediated by AhR-independent p38 activation in intestinal epithelial Caco-2 cells [79]. Conversely, the precise function of skatole in the regulation of IL-6 expression, which is crucial for chronic inflammation, along with TNF-α, remains unclear. The focus on skatole in the present study may provide novel insights into the mechanisms linking diet, gut microbiota metabolites, and chronic inflammation in relation to IBD and CRC. Furthermore, novel targets for intervention against chronic inflammation associated with IBD and CRC should be identified. Hence, the present study aimed to elucidate how skatole regulates IL-6 and TNF-α expression by focusing on the NF-κB activation mechanism. Specifically, our focus was on the effects of skatole-induced activation of mitogen-activated protein kinases (MAPKs) such as extracellular signal-regulated kinases (ERKs), p38, and c-Jun N-terminal kinase (JNK), and AhR, which we have previously established in our research [78,79], on NF-κB activation.

## 2. Materials and Methods

### 2.1. Materials

The following reagents and antibodies were sourced from various suppliers: Anti-β-actin (C4) was obtained from Santa Cruz Biotechnology Inc. (Dallas, TX, USA). Cell Signaling Technology Inc. (Danvers, MA, USA) supplied anti-IL-6 (D3K2N), anti-phospho-NF-κB p65 (Ser276), anti-phospho-p44/42 MAPK (Erk1/2) (Thr202/Tyr204), anti-phospho-MAPKAPK-2 (Thr334) (27B7), and anti-phospho-c-Jun (Ser73) (D47G9) XP^®^. Jackson ImmunoResearch Laboratories, Inc. (West Grove, PA, USA) supplied Peroxidase AffiniPure Goat Anti-Rabbit IgG (H + L) and Anti-Mouse IgG (H + L) antibodies. Nacalai Tesque Inc. (Kyoto, Japan) furnished Protease Inhibitor Cocktail (EDTA-free) (100×) and Phosphatase Inhibitor Cocktail. 3-Methylindole (skatole) (>98.0%(GC)) was procured from Tokyo Chemical Industry Co., Ltd. (Tokyo, Japan). Wako Pure Chemical Industries, Ltd. (Osaka, Japan) provided penicillin–streptomycin solution (×100), Dulbecco’s modified Eagle’s medium (DMEM) with high glucose, BAY 11-7082 (NF-κB inhibitor), U0126 (ERK pathway inhibitor; mitogen-activated protein kinase kinase (MEK) 1/2 inhibitor), SB203580 (p38 inhibitor), and SP600125 (JNK inhibitor). CH223191 (AhR antagonist) was acquired from Cayman Chemical (Ann Arbor, MI, USA). Fetal bovine serum (FBS) was purchased from Biowest S.A.S. (Nuaillé, France).

### 2.2. Cell Culture

Caco-2 cells, a human intestinal epithelial cell line, were acquired from RIKEN Cell Bank (Tsukuba, Japan). Cells were cultured according to previously established methods [78,79]. Before conducting any experiments, the cells were maintained in serum-free DMEM for 24 h. DMSO served as both the control and vehicle for various compounds including skatole, CH223191, BAY 11-7082, U0126, SB203580, and SP600125. The final DMSO concentration was 0.1% (*v*/*v*).

### 2.3. Cell Viability

Intestinal epithelial Caco-2 cells were exposed to either vehicle (0.1% DMSO; control) or BAY 11-7082 (10 μM) for 60 min. Subsequently, the cells were treated with either vehicle (0.1% DMSO; control) or varying concentrations of skatole for 24 h. Cell viability was assessed using the Cell Counting Kit-8 (Dojindo, Kumamoto, Japan), following a previously described method [70].

### 2.4. Transfection and Luciferase Assays

To evaluate the promoter activity of TNF-α and IL-6 as well as the transcriptional activity of NF-κB and AhR in intestinal epithelial Caco-2 cells, various plasmids were used. These included pGL4-phTNF and pGL4-phIL6 (both from RIKEN Bio-Resource Center, Tsukuba, Japan), pGL4.32[luc2p/NF-κB-RE/Hygro] and pGL4.43[luc2P/XRE/Hygro] (both from Promega, Madison, WI, USA), and pRL-SV40 plasmids (Promega, Madison, WI, USA). The plasmids were introduced into cells using FuGENE HD (Roche, Mannheim, Germany). Following a 24-h incubation period, the transfected intestinal epithelial Caco-2 cells were exposed to either vehicle (0.1% DMSO; control) or skatole (1000 μM) for 6 h. Subsequent measurements were performed using a Junior LB9509 luminometer (Berthold Technologies, Bad Wildbad, Germany) using previously reported methods [70,78]. The values represent the replicate averages of three independent experiments.

### 2.5. Quantitative Real-Time PCR

Intestinal epithelial Caco-2 cells were subjected to various treatments. These included vehicle (0.1% DMSO; control), CH223191 (10 μM), U0126 (5 μM), SB203580 (10 μM), and SP600125 (5 μM) for 30 min, or vehicle (0.1% DMSO; control) and BAY 11-7082 (10 μM) for 60 min. Subsequently, the cells were stimulated with either vehicle (0.1% DMSO; control) or skatole (1000 μM) for various durations. RNA was extracted from intestinal epithelial Caco-2 cells, and 1 μg of this RNA was used to synthesize first-strand cDNA using previously described techniques [78,79]. Quantitative real-time PCR was subsequently performed using a Thermal Cycler Dice Real Time System III (Takara Bio Co., Inc., Kusatsu, Japan), following the methods outlined in earlier research [80]. The oligonucleotide primers used are listed in Table 1. To quantify the amplicons, a calibration curve was generated using serially diluted DNA samples by plotting cycle threshold (Cq) values against the logarithm of sample concentration. mRNA expression was determined by calculating the ratio relative to the mRNA of the human ribosomal protein lateral subunit P0 (*RPLP0*), which served as an internal standard.

### 2.6. Immunoblotting

Intestinal epithelial Caco-2 cells were exposed to either a control solution (0.1% DMSO), various antagonists, or inhibitors prior to treatment with either the control solution or 1000 μM of skatole for various durations. The proteins in the medium were concentrated by adding ice-cold acetone or the cells were lysed using lysis buffer as previously described [70,79]. Protein samples were separated using sodium dodecyl sulfate-polyacrylamide gel electrophoresis and subsequently transferred to Immobilon-P polyvinylidene fluoride membranes (Millipore Inc., Bedford, MA, USA). The membranes were probed with antibodies against IL-6 (1:1000), phospho-NF-κB p65 (S276) (1:1000), phospho-ERK1/2 (Thr202/Tyr204) (1:5000), phospho-MAPKAPK-2 (Thr334) (1:1000), phospho-c-Jun (Ser73) (1:1000), and β-actin (1:5000). Visualization of the target proteins was accomplished utilizing the Chemi-Lumi One L or Chemi-Lumi One Super system (Nacalai Tesque Inc., Kyoto, Japan), followed by analysis with an ImageQuant LAS 4010 densitometer (GE Healthcare Life Sciences, Uppsala, Sweden). Densitometric measurements of the blotted proteins were conducted utilizing Image J 1.53k software with the Band/Peak Quantification macro [81]. The levels of phosphorylated proteins were normalized to β-actin and expressed as fold increases relative to the control.

### 2.7. Statistical Analysis

**Figure 1:**
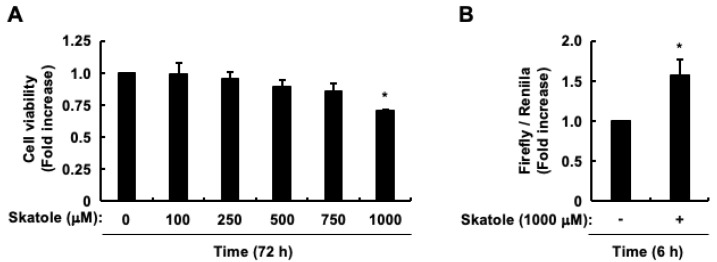
Impact of skatole on intestinal epithelial Caco-2 cells: assessment of cell viability and TNF-α promoter activity. (**A**) Cell viability. Data represent the mean ± SE of quadruplicates from n = 3. * *p* < 0.05 vs. vehicle (DMSO). (**B**) TNF-α promoter activity. Data represent the mean ± SE of duplicates from n = 3. * *p* < 0.05 vs. vehicle (DMSO).

**Figure 2:**
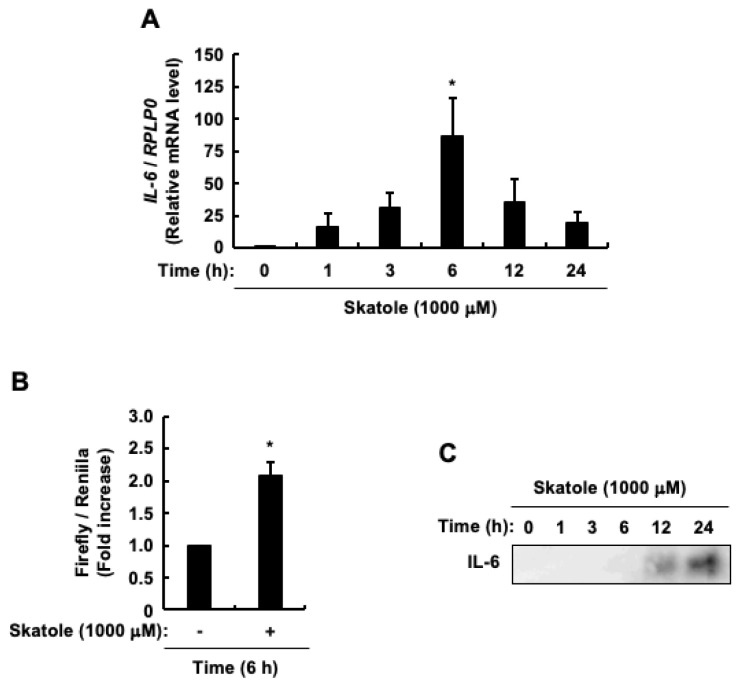
Impact of skatole on interleukin-6 (IL-6) gene expression and promoter function in intestinal epithelial Caco-2 cells. (**A**) *IL-6* mRNA expression. Data are expressed as the mean ± SE of n = 3. * *p* < 0.05 vs. 0 h. (**B**) IL-6 promoter activity. Data represent the mean ± SE of duplicates from n = 3. * *p* < 0.05 vs. vehicle (DMSO). (**C**) IL-6 protein.

**Figure 3:**
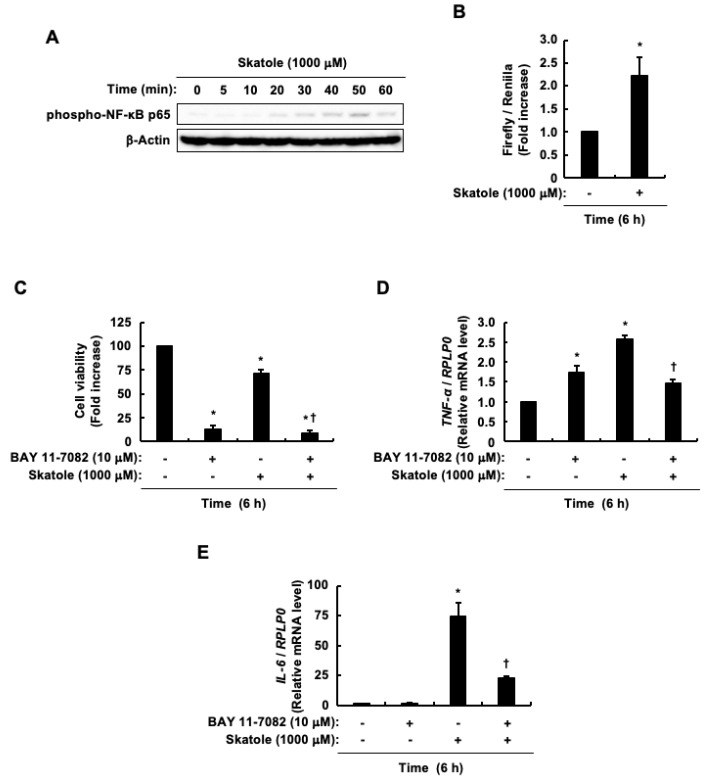
Impact of NF-κB on skatole-triggered cell death and the expression of *TNF-α* and *IL-6* mRNA in intestinal epithelial Caco-2 cells. (**A**) Phospho-NF-κB p65 (Ser276) and β-actin. (**B**) NF-κB transcriptional activity. Data represent the mean ± SE of duplicates from n = 3. * *p* < 0.05 vs. vehicle (DMSO). (**C**) Cell viability. Data represent the mean ± SE of quadruplicates from n = 3. * *p* < 0.05 vs. vehicle (DMSO). ^†^ *p* < 0.05 vs. skatole alone. (**D**) *TNF-α* mRNA expression. Data are expressed as the mean ± SE of n = 3. * *p* < 0.05 vs. vehicle (DMSO). ^†^ *p* < 0.05 vs. skatole alone. (**E**) *IL-6* mRNA expression. Data are expressed as the mean ± SE of n = 3. * *p* < 0.05 vs. vehicle (DMSO). ^†^ *p* < 0.05 vs. skatole alone.

**Figure 4:**
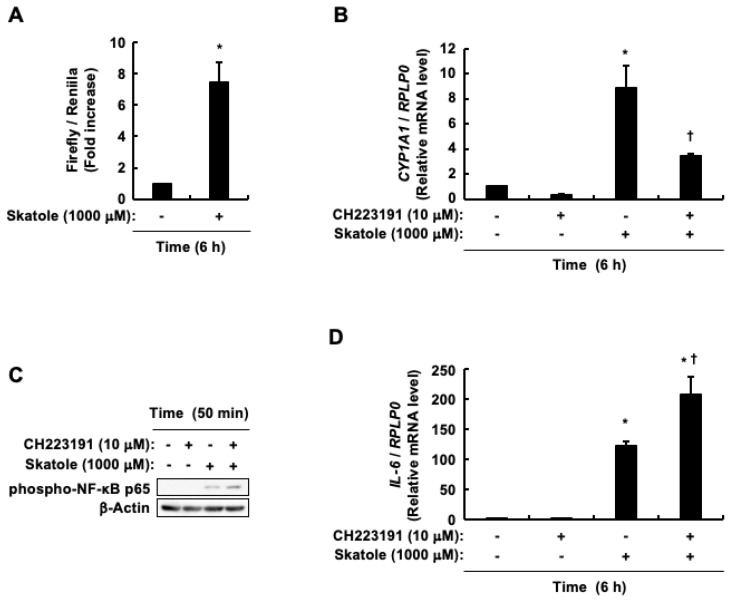
Impact of AhR on the NF-κB phosphorylation and *IL-6* mRNA expression triggered by skatole in intestinal epithelial Caco-2 cells. (**A**) AhR transcriptional activity. Data represent the mean ± SE of duplicates from n = 3. * *p* < 0.05 vs. vehicle (DMSO). (**B**) *CYP1A1* mRNA expression. Data are expressed as the mean ± SE of n = 3. * *p* < 0.05 vs. vehicle (DMSO). ^†^ *p* < 0.05 vs. skatole alone. (**C**) Phospho-NF-κB p65 (Ser276) and β-actin. (**D**) *IL-6* mRNA expression. Data are expressed as the mean ± SE of n = 3. * *p* < 0.05 vs. vehicle (DMSO). ^†^ *p* < 0.05 vs. skatole alone.

**Figure 5:**
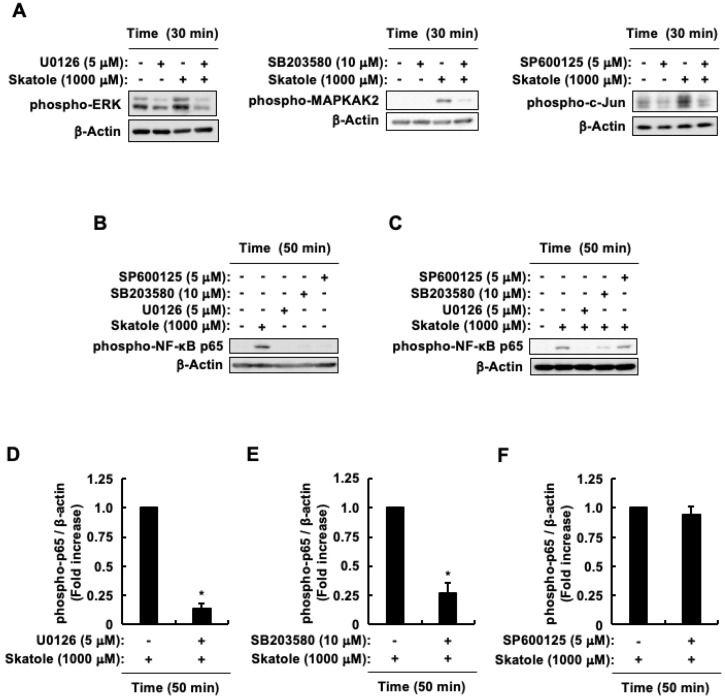
Impact of MAPKs on NF-κB activation triggered by skatole in intestinal epithelial Caco-2 cells. (**A**) Phospho-ERK, phospho-MAPKAPK2, phospho-c-Jun, and β-actin. (**B**,**C**) Phospho-NF-κB p65 (Ser276) and β-actin. Effect of U0126 (**D**), SB203580 (**E**), and SP600125 (**F**) on phosphorylation levels of phospho-NF-κB p65 (Ser276). Band intensity of phospho-NF-κB p65 (Ser276) normalized to β-actin. Data are expressed as the mean ± SE of n = 3. * *p* < 0.05 vs. skatole alone.

**Figure 6:**
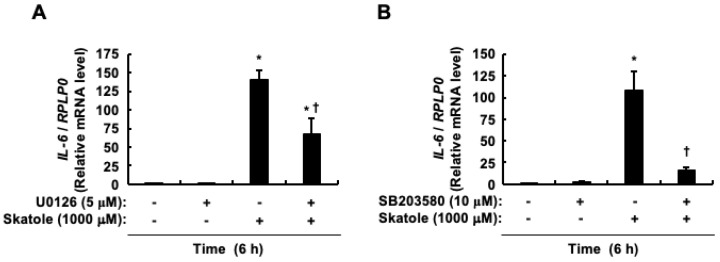
Impact of ERK and p38 on *IL-6* mRNA expression induced by skatole in intestinal epithelial Caco-2 cells. Effect of U0126 (**A**) and SB203580 (**B**) on *IL-6* mRNA expression. Data are expressed as the mean ± SE of n = 3. * *p* < 0.05 vs. vehicle (DMSO). ^†^ *p* < 0.05 vs. skatole alone.

Data are expressed as mean ± standard error (SE). Statistical analysis was conducted using Dunnett’s test (Figure 1A and Figure 2A), the Tukey–Kramer test (Figure 3C–E, Figure 4B,D, Figure 5A,B and Figure 6A,B), and Student’s *t*-test (Figure 1B, Figure 2B, Figure 3B, Figure 4A and Figure 5D–F). These analyses were performed using Excel 2011 (Microsoft Corp., Redmond, WA, USA) and Statcel 4 software (OMS Publishing Co., Saitama, Japan). Statistical significance was set at *p* < 0.05.

## 3. Results

### 3.1. Skatole Induces Cell Death in Caco-2 Intestinal Epithelial Cells and Plays a Role in Regulating TNF-α Expression, Aligning with Previous Findings

The molar concentration of skatole in feces is estimated to be approximately 1000 μM [73,82], based on several factors: skatole’s molecular weight of 131.17, its concentration in feces of approximately 100 μg/g in patients with CRC [72] and those with impaired intestinal digestion [73,74], a fecal density averaging 1.07 g/mL (range 1.06–1.09), and an average water content of 75% (range 63–86). In line with previous studies [78,79], the present study utilized skatole concentrations of up to 1000 µM, representing the maximum level. Given that intestinal epithelial Caco-2 cells are considered a suitable model for investigating cytokine secretion regulation by intestinal epithelial cells (IECs) [78], and that our prior research examined skatole-induced regulation of TNF-α expression in these cells [79], we employed them in the present study. We initially verified that the Caco-2 cells used here exhibited characteristics similar to those in our previous studies [78,79], particularly regarding cell viability and TNF-α expression regulation. Figure 1A demonstrates that 1000 μM skatole led to significant cell death, consistent with our earlier findings [78,79]. Additionally, although we previously reported the effect of skatole on *TNF-α* mRNA and protein levels, we did not analyze its impact on TNF-α promoter activity. Our current study revealed a significant increase in this activity after 6 h (Figure 1B). Our findings align with those of our previous studies [78,79], confirming the reliability of our experimental conditions.

### 3.2. Skatole Elevates the Promoter Activity of IL-6 as Well as TNF-α and Increases IL-6 mRNA and Protein Expression

Given that skatole increased TNF-α promoter activity (Figure 1B) as well as mRNA and protein levels of TNF-α in intestinal epithelial Caco-2 cells [79], the present study examined the effects of skatole on the regulation of IL-6 expression. The mRNA expression of *IL-6* was significantly upregulated after 6 h (Figure 2A). Similarly, the IL-6 promoter activity exhibited a marked increase at identical time points (Figure 2B). The culture medium revealed the presence of secreted IL-6 protein between 12 h and 24 h (Figure 2C). These findings suggest that skatole is the driving force behind increased IL-6 promoter activity, mRNA expression, and protein secretion. Because of the correlation among these three results, further analysis was conducted using *IL-6* mRNA expression levels as a marker, employing the highly quantitative method of real-time PCR.

### 3.3. NF-κB Activation Is Induced by Skatole and Contributes to Intestinal Epithelial Caco-2 Cell Survival and Increased IL-6 and TNF-α Expression

Our previous research demonstrated that skatole activates AhR and MAPKs in intestinal epithelial Caco-2 cells [78,79]; however, its effect on NF-κB remains unclear. The present study investigated whether skatole could trigger the phosphorylation of serine 276 in NF-κB p65, a process linked to NF-κB p65 activation. Its phosphorylation commenced 20 min after skatole exposure, peaked at 50 min, and started to decline at 60 min (Figure 3A). We also evaluated NF-κB transcriptional activity and found that skatole significantly elevated NF-κB transcriptional activity (Figure 3B). These findings indicate that skatole-induced phosphorylation of serine 276 in NF-κB p65 increases its transcriptional activity. Consequently, subsequent experiments utilized the phosphorylation of serine 276 in NF-κB p65 as an indicator of NF-κB activation. We examined the relationship between skatole-induced cell death in intestinal epithelial Caco-2 cells and NF-κB activation. Figure 3C shows that BAY 11-7082, an NF-κB inhibitor, considerably increased cell death in the presence or absence of skatole. Given that skatole-induced *TNF-α* mRNA and protein levels were previously shown to correlate [79], we analyzed *TNF-α* mRNA levels as an indicator, in the same way as IL-6. BAY 11-7082 reduced the skatole-induced increase in *TNF-α* mRNA expression (Figure 3D). Moreover, BAY 11-7082 also reduced the skatole-induced elevation of *IL-6* mRNA levels (Figure 3E). Collectively, these results suggest that NF-κB plays a significant role in the survival of intestinal epithelial Caco-2 cells and skatole increases IL-6 and TNF-α expression by activating NF-κB. Additionally, since the skatole-induced increase in IL-6 expression was not completely repressed by BAY 11-7082, signal transduction molecules other than NF-κB may also regulate skatole-induced increases in IL-6 expression.

### 3.4. Activation of AhR Partially Mitigates the Increase in IL-6 Expression Caused by Skatole by Attenuating the Activation of NF-κB

Consistent with our earlier findings [78,79], skatole triggered an increase in the transcriptional activity of AhR (Figure 4A). Additionally, CH223191, an AhR antagonist, partially suppressed the skatole-mediated increase in mRNA expression of its typical target gene, *CYP1A1* (Figure 4B). These observations confirmed CH223191’s effectiveness as an AhR antagonist, while also suggesting that skatole-induced elevation in CYP1A1 expression may be regulated by signaling molecules other than AhR. Building on these results and our previous report that CH223191 enhances skatole-induced TNF-α expression [79], we investigated whether AhR contributes to the NF-κB activation and increased IL-6 expression induced by skatole. Figure 4C shows that CH223191 amplified the activation of NF-κB in response to skatole and intensified the skatole-induced increase in *IL-6* mRNA expression (Figure 4D). Taken together, the activation of AhR induced by skatole suggests that it partially suppresses the increase in IL-6 expression, similar to TNF-α [79]. This may be, at least in part, attributed to the attenuated NF-κB activation.

### 3.5. Activation of ERK and p38 Involved in the NF-κB/IL-6 Pathway by Skatole, but Not JNK

We not only examined the connection between the activation of MAPKs and elevated TNF-α expression [79] but also explored the relationship between NF-κB activation and increased TNF-α expression (Figure 3E). To further investigate the pathways involving MAPK activation, NF-κB activation, and increased IL-6 expression caused by skatole, we validated the effects of specific inhibitors: U0126 for the ERK pathway, SB203580 for p38, and SP600125 for JNK. As shown in Figure 5A, U0126 successfully blocked ERK activation in response to skatole stimulation. Similarly, SB203580 and SP600125 inhibited the phosphorylation of MAPKAPK2 and c-Jun, which are the downstream targets of p38 and JNK, respectively. Based on these observations, we assessed the potential influence of skatole-induced ERK, p38, and JNK activation on NF-κB activation. Figure 5B demonstrates that skatole triggered the phosphorylation of NF-κB p65, whereas individual treatments with U0126, SB203580, or SP600125 did not affect its phosphorylation status. However, when these three inhibitors were administered prior to skatole stimulation, U0126 and SB203580 successfully prevented skatole-induced NF-κB p65 phosphorylation (Figure 5C–E), whereas SP600125 had no effect (Figure 5C,F). Furthermore, Figure 6A,B illustrates that both U0126 and SB203580 effectively but partially reduced the skatole-induced increase in *IL-6* mRNA expression. Collectively, these results suggest that skatole triggers NF-κB activation through the stimulation of the ERK and p38 pathways, ultimately resulting in enhanced IL-6 expression. Furthermore, the increase in IL-6 expression induced by skatole was not completely suppressed by U0126 and SB203580, in addition to BAY 11-7082, suggesting that signaling molecules other than ERK, p38, and NF-κB may also regulate the increase in IL-6 expression induced by skatole.

## 4. Discussion

**Figure 7:**
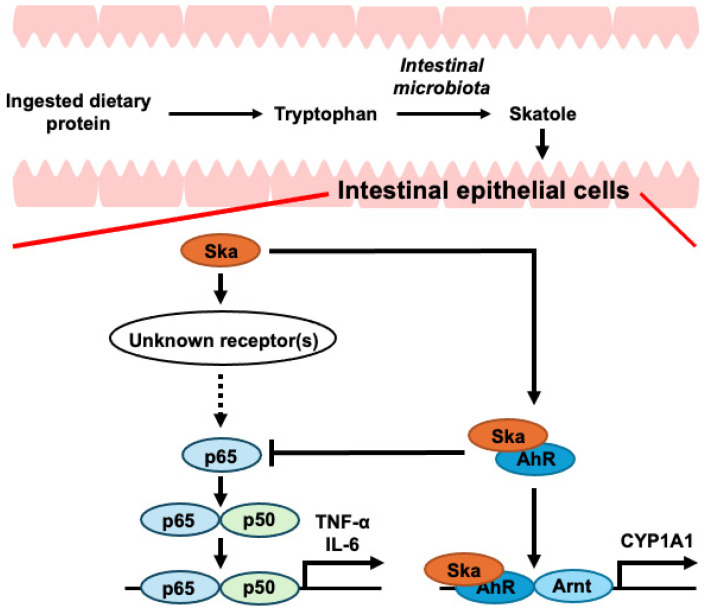
Diagrammatic representation illustrating the regulatory pathways of skatole-induced IL-6 and TNF-α expression. Skatole stimulates NF-κB, resulting in enhanced production of IL-6 and TNF-α. While AhR amplifies skatole-induced CYP1A1 expression, it partially inhibits both skatole-triggered NF-κB activation and upregulation of IL-6 and TNF-α expression. This suggests that IL-6 and TNF-α production may be regulated by the interplay between NF-κB and AhR activation. These findings indicate that skatole may contribute to the onset and progression of inflammatory bowel disease (IBD) and colorectal cancer (CRC) through increased IL-6 and TNF-α expression. Furthermore, our study implies that activated AhR modulates skatole-induced IL-6 and TNF-α expression, potentially influencing IBD and CRC development and progression. AhR, aryl hydrocarbon receptor; Arnt, aryl hydrocarbon receptor nuclear translocator; CYP1A1, cytochrome P450 1A1; IL-6, interleukin-6; p65, nuclear factor-κB (NF-κB) p65; p50, NF-κB p50; XRE, xenobiotic response element.

Although skatole-induced JNK activation did not trigger NF-κB activation, skatole-mediated activation of ERK and p38 led to NF-κB activation. This NF-κB activation is involved in the skatole-mediated upregulation of IL-6 and TNF-α expression in colonic epithelial cells under pathological conditions, including IBD and CRC. Furthermore, CH223191 enhanced the NF-κB activation and increased IL-6 expression induced by skatole, as well as increasing TNF-α expression triggered by skatole, as previously reported [79]. This suggests that the skatole-induced increases in IL-6 and TNF-α expression are regulated by the balance between the strength of activation of NF-κB and that of AhR. However, further studies, including animal studies, are required to translate the new findings shown in Figure 7 obtained from these cellular models into a deeper understanding of the roles of IBD and CRC progression in clinical practice.

Similar to our previous report, skatole also induced cell death in intestinal epithelial Caco-2 cells [78,79]. We have shown that part of this cell death is mediated by the increase in TNF-α expression induced by skatole [79]. Although this result is consistent with the promotion of IBD progression, it presents a contradiction in that elevated TNF-α expression is associated with CRC proliferation [52] and the progression of advanced CRC [54]. Similar contradictions are observed in the case of bile acids, which have been associated with the advancement of IBD and CRC [83,84,85]. Elevated concentrations of bile acids in the colon induce mitotic disruption and stimulate the promotion of epithelial apoptosis [D1, D2]. Furthermore, extended contact with bile acids leads to apoptosis resistance and stimulates cell proliferation [83,84]. Indeed, in rats exposed to γ-irradiation-induced DNA damage, IECs resistant to apoptosis exhibit growth and viability when exposed to cholic and deoxycholic acids, along with their related bile acids found in the digestive system [86]. Considering that significant amounts of skatole are generated within the large intestine of patients with CRC [72], this may contribute to the advancement of CRC through mechanisms similar to those of bile acids.

Given that skatole triggers cell death through increased TNF-α expression [79] and that skatole-activated NF-κB leads to increased TNF-α expression, we predicted that BAY 11-7082 would inhibit skatole-induced cell death. Contrary to our prediction, BAY 11-7082 alone decreased the survival rate of intestinal epithelial Caco-2 cells, and the same was observed under skatole stimulation. Based on the present findings, the decreased survival of intestinal epithelial Caco-2 cells with BAY 11-7082 alone is considered be due to the increased TNF-α expression induced by BAY 11-7082 treatment alone. On the other hand, intestinal epithelial Caco-2 cells exposed to IL-6 are reported to induce a substantial increase in secretory clusterin production, which promotes tumor cell survival, in addition to interfering with Bax-mediated cell death by suppressing the apoptosis-promoting function of Bax [87]. Thus, NF-κB activation induced by skatole may have a greater impact on viability and proliferative capacity, including increased IL-6 expression, than on cell death by increased TNF-α expression in intestinal epithelial Caco-2 cells.

The current investigation demonstrated that CH223191 amplifies the skatole-induced increase in IL-6 expression. Similarly, we reported that the increased TNF-α expression induced by skatole is also promoted by CH223191 [79]. These observations imply that the inhibitory mechanism of AhR against the skatole-induced increases in IL-6 and TNF-α expression may not function optimally when skatole acts on the intestinal mucosa of patients with IBD, in a manner similar to its effect on intestinal epithelial Caco-2 cells. Considering that people with IBD have lower AhR expression in their intestinal mucosa compared to healthy individuals [88], it is plausible that skatole could more significantly elevate IL-6 and TNF-α expression in those with IBD. However, AhR expression is higher in CRC tissues than in normal colon tissues [89,90,91]. Additionally, increased AhR expression has been observed in EpCAM+ epithelial cells isolated from CRC [92]. Thus, the ability of skatole to enhance IL-6 and TNF-α expression in colonic tissues may be attenuated compared with that observed in healthy individuals or those with IBD. Additionally, our findings suggest that drugs, dietary components, and gut microbiota metabolites antagonistic to AhR could contribute to IBD and CRC progression by increasing skatole-induced IL-6 and TNF-α expression. Although AhR activation is reported to suppress increased TNF-α expression, the mechanism underlying this suppression has not yet been elucidated [93]. On the other hand, the process through which AhR activation suppresses the increased IL-6 expression is reported as follows. 7,12-dimethylbenz[*a*]anthracene (DMBA) and 2,3,7,8-tetrachlorodibenzo-*p*-dioxin (TCDD) have been demonstrated to activate AhR in BMS2 cells, a bone marrow stromal cell line [94,95]; both AhR ligands suppress the LPS-induced increase in IL-6 expression [96]. This mechanism appears to be regulated by AhR-mediated signaling, which in turn suppresses NF-κB transcriptional activity [96]. In the present study, since skatole itself activates both AhR and NF-κB, the suppression of AhR signaling using CH223191 may enhance the increase in IL-6 and TNF-α expression associated with the elevation of NF-κB transcriptional activity through a mechanism similar to that reported above [68]. In contrast, indoxyl sulfate, an indole derivative similar to skatole, induces the elevation of NF-κB transcriptional activity by binding to AhR, leading to an increase in IL-6 and TNF-α expression in vascular cells [97,98]. Taken together, these findings suggest that AhR activation influences NF-κB transcriptional activity, and that IL-6 and TNF-α expression are more likely to be influenced by cell type than ligand type.

Current research indicates that inhibiting only the ERK or p38 pathways prevents the phosphorylation of NF-κB p65 in response to skatole. These findings imply that there is no direct interaction between NF-κB p65 and ERK or p38. Indeed, at least for p38, there is evidence that it does not directly phosphorylate NF-κB p65 [60]. Therefore, the phosphorylation of NF-κB p65 caused by skatole is likely mediated by molecules that are activated by EKR and p38. Based on previous reports, mitogen- and stress-activated protein kinase-1 (MSK1), known to be activated by ERK and p38, is potentially involved in the skatole-induced phosphorylation of NF-κB p65 [63].

The present study demonstrated that BAY 11-7082 partially suppressed the skatole-induced increase in TNF-α expression, but treatment with a BAY 11-7082 inhibitor alone also led to increased TNF-α expression. Additionally, U0126 inhibited skatole-induced NF-κB activation. However, U0126 did not influence the skatole-induced increase in TNF-α expression [79]. Considering these results, molecules whose activity was suppressed by activated ERK and NF-κB may be activated by the decrease in ERK and NF-κB activity mediated by U0126. Based on this prediction, the effects of skatole-induced ERK and NF-κB activation on the regulation of TNF-α expression are considered to be complex. Therefore, the identification of signaling pathways downstream of ERK and NF-κB activated by skatole, which are still unresolved, is expected to further our understanding of the mechanism of skatole action on the increase in TNF-α expression.

Suppressing ERK activation in the intestinal cells and intestinal epithelial Caco-2 cells of IL-6 knockout mice increases their MUC3 expression [99,100]. The function of mucins in the intestine is to attenuate the action of 3-oxo-C_12_-HSL generated by certain species of bacteria, such as *Pseudomonas aeruginosa* [101,102]. In addition, the activation of ERK induced by 3-oxo-C_12_-HSL enhances its own action on target cells by downregulating MUC3 expression [102]. Taken together, these findings suggest that the activation of the ERK/IL-6 pathway by skatole leads to a decrease in MUC3 expression. This reduction in MUC3 expression may increase the sensitivity of both IECs and CRC cells to skatole, thereby leading to an increase in IL-6 and TNF-α expression.

## 5. Conclusions

Skatole leads to NF-κB activation, culminating in increased IL-6 and TNF-α expression. This process appears to be mediated by specific receptors that remain unidentified. Further research is required to elucidate these mechanisms. Moreover, the AhR activation induced by skatole partially suppresses the increase in IL-6 and TNF-α expression associated with NF-κB activation. Based on these results, the extent to which IL-6 and TNF-α expression are upregulated by skatole may be determined by the equilibrium between the signaling strength of NF-κB and AhR activation. In addition to skatole accumulation in the feces of patients with CRC [72,73], IAA inhibits the proliferation and inflammatory response of CRC cells [70,71], suggesting that a potential new therapeutic approach for CRC and IBD could involve preventing the conversion of IAA to skatole in the colon, thereby increasing the IAA levels. Therefore, our future objective will be to identify food components and develop drugs that exert inhibitory effects on indoleacetate decarboxylase, which is involved in the metabolism of IAA to skatole [103]. Furthermore, we will also attempt to identify a new target receptor for skatole that induces NF-κB activation, which will lead to the development of its antagonist. Such findings may contribute to the treatment of CRC and IBD and potentially inhibit their pathological progression.

## Figures and Tables

**Table 1:** Forward (Fw) and reverse (Rv) primers for target genes.

Target Genes	GenBank Accession No.	Primers (5→3′)	Length (bp)	Product Length (bp)
*IL-6*	NM_000600.5	Fw: CCTGAACCTTCCAAAGATGGC	21	75
		Rv: TTCACCAGGCAAGTCTCCTCA	21	
*TNF-α*	NM_000594	Fw: GAGGCCAAGCCCTGGTATG	19	91
		Rv: CGGGCCGATTGATCTCAGC	19	
*CYP1A1*	NM_000499.5	Fw: TTCTGAACTGCAAGTGCTGCAT	20	94
		Rv: ATCTGCTGCATCTGCTTG	20	
*RPLP0*	NM_001002.4	Fw: CGACCTGGAAGTCCAACTAC	20	108
		Rv: ATCTGCTGCATCTGCTTG	18	

## Data Availability

The data presented in this study are available on request from the corresponding author.
